# Keep Your Finger on the Oxygen Pulse When Interpreting Exercise Hemodynamics and Prognosis in HFpEF

**DOI:** 10.1016/j.jacadv.2024.101097

**Published:** 2024-08-28

**Authors:** Marco Guazzi

**Affiliations:** Department of Cardiology, San Paolo Hospital, University of Milano School of Medicine, Milano, Italy

**Keywords:** exercise, HFpEF, oxygen pulse

**Figure:**
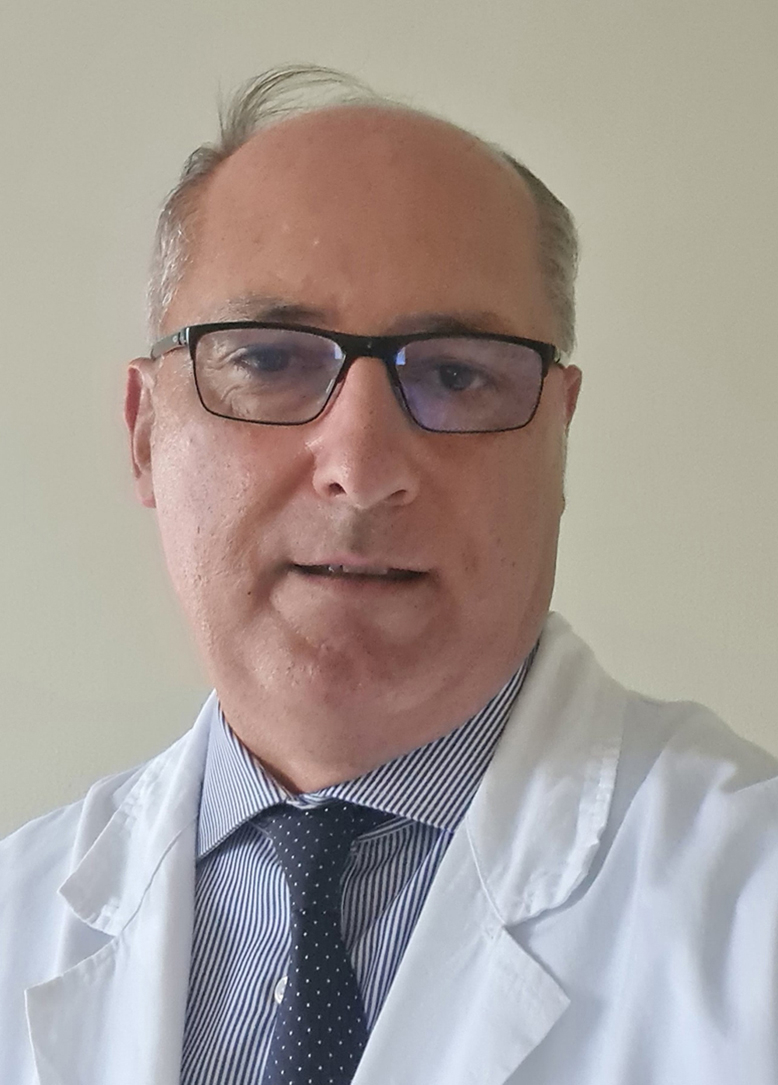

Since its introduction in clinical practice, more than 40 years ago, gas exchange analysis by cardiopulmonary exercise testing (CPET) has become the gold standard for assessing exercise limitation of heart failure (HF) patients, providing information on the organ systems involved in the limiting steps of oxygen uptake ($\dot{\text{V}}{\text{O}}_{2}$) cascade.^1^ HF, of whatever origin and left ventricular ejection fraction, is the typical example of how many factors, that is, circulatory, pulmonary, metabolic, or peripheral, are involved in the loss of aerobic capacity. The relative implications of each organ system involved may be precisely scrutinized by the integrated interpretation of the many respective physiological variables recorded by gas exchange analysis.^2^

Paradoxically, although gas exchange offers a true potential for deepening into the cardiorespiratory pathophysiology, the majority of clinical studies primarily focus on the 2 main CPET-derived variables, $\dot{\text{V}}{\text{O}}_{2}$ at peak exercise^3^ and the breathing efficiency assessed by the slope of incremental ventilation (VE) vs carbon dioxide production (VCO_2_),^4^ considering their highly prognostic prediction in reflecting the final downstream paths involved in the impaired cardiac reserve response. Over time, peak $\dot{\text{V}}{\text{O}}_{2}$ and VE/VCO_2_ slope have become cornerstone in the classification of disease severity^5^ with their incorporation in scores predictive of risk,^6^ now broadly utilized as endpoints of interventional trials.

Historically, the advancements in the interpretation of gas exchange pathophysiology have been driven by a large number of studies performed in HF patients with reduced ejection fraction but most recently the increasing numbers of reports combining CPET with imaging techniques (mainly echocardiography)^7^ or invasive CPET with direct measures of cardiac output (CO) and arteriovenous (a-v) O_2_ difference^8^ have seen a broader applications of exercise gas exchange to the garden variety of HF with preserved ejection fraction (pEF) phenotypes.

In this issue of *JACC: Advances*, Li et al^9^ offer some important steps forward in the field presenting the results of a proof-of-concept study of HFpEF patients undergoing invasive CPET. Based on the strong physiological background they advanced the hypothesis that O_2_ pulse, a landmark but underused CPET-derived variable, may correlate with the left and right ventricular-vascular uncoupling investigated through an array of invasively determined measures such as the proportionate pulse pressure, arterial elastance, mean pulmonary arterial pressure/CO, right ventricular stroke volume (SV) index, and pulmonary arterial wedge pressure/CO. They also postulated that in HFpEF O_2_ pulse may overcome $\dot{\text{V}}{\text{O}}_{2}$ and VE efficiency as comparators for outcome prediction. O_2_ pulse is the ratio of $\dot{\text{V}}{\text{O}}_{2}$ to heart rate indicating the amount of O_2_ extracted per heart beat.^2^ As such it reflects the whole determinants of the Fick principle and provides an estimate of left ventricular SV changes for a given heart rate during exercise, assuming that the (a-v) O_2_ is maximal. Authors focused on O_2_ pulse corrected for percent predicted (% Pred) normal value, subgrouping the population in tertiles with the highest one performing at the best level of O_2_ kinetic pattern, that is, highest peak $\dot{\text{V}}{\text{O}}_{2}$, SV, and (a-v) O_2_ difference. No differences across tertile groups were observed in terms of HR response, which excludes the potential that HR, the other determinant of CO increase during maximal exercise, would play a contributory role to peak $\dot{\text{V}}{\text{O}}_{2}$ differences. When performing a sensitive and specificity analysis, a cutoff % Pred O_2_ pulse >85% documented a 100% concordance rate of % pred SV. As expected, subjects with the most efficient aerobic performance exhibited also the best VE efficiency.

Of note, % Pred O_2_ pulse outperformed the traditional pillars of CPET-derived prognostic marker such as peak $\dot{\text{V}}{\text{O}}_{2}$ and VE/VCO_2_ slope and was a strong independent marker of all-cause mortality when adjusted for peak $\dot{\text{V}}{\text{O}}_{2}$ and age or VE/VCO_2_ slope and age.

Finally, % Pred O_2_ pulse correlated with measures of exercise left and right ventricular-vascular coupling.

So, how these findings may help to expand our vision on the role of exercise gas exchange analysis in the management of HFpEF? Out of the powerful prognostic role of % Pred O_2_ pulse, could these data refine our perspectives on a gas exchange-driven approach to HFpEF, perhaps in a continuum from its diagnosis to treatment strategies?

Data stress the concept that a broadest multiparametric approach of CPET might definitively impact on the diagnostic algorithms of HFpEF. Peak $\dot{\text{V}}{\text{O}}_{2}$ is sensitive but not specific in patients with unexplained dyspnea and subsequent diagnosis of HFpEF,^8^ while VE/VCO_2_ slope is specific but less sensitive with highest slopes observed in the most advanced stages of HFpEF associated with pulmonary hypertension and pulmonary vascular disease.^10^, ^11^, ^12^ For these reasons, the Mayo Clinic heavy, hypertensive [H2], atrial fibrillation [F], pulmonary hypertension [P], elder [E], filling pressure [F] diagnostic score for HFpEF does not incorporate CPET-derived variables and the Heart Failure Association- [P: pre-test assessment], [E: echocardiography], [F1: functional testing], [F2: final aetiology] score prospects a role for gas exchange analysis just for ruling a noncardiac-related impairment. It follows that a search for most sensitive and specific variable such as % Pred O_2_ pulse could ease the prioritization of CPET within the diagnostic scores.^13^

This is the only study addressing the concordance between % Pred O_2_ pulse and invasively measured % Pred SV that is optimal in the presence of a high pattern of performance and for high % Pred SV but for intermediate and lower ranges of % Pred O_2_ pulse, the (a-v) O_2_ fraction acquires a contributory role. These informative directions are novel revealing in which case the third component of the Fick principle, that is, O_2_ extraction, should be directly measured rather than assumed as normal.

Under this respect although O_2_ pulse may be confirmed as an attractive and reliable measure of SV, some caution is needed in patients whose O_2_ extraction is not ideal such as anemic patients or those in whom the expected linearity in the increase of exercise $\dot{\text{V}}{\text{O}}_{2}$ and CO may not reasonably be made. Correlations of % Pred O_2_ pulse with indicators of left and right ventricular-vascular uncoupling are intriguing and worth of future confirmation in methodological studies of comparison with other noninvasive estimates of biventricular coupling in use in clinical practice.

Out of the potential aforementioned caveat, % Pred O_2_ pulse should receive attention also in the assessment of therapeutic benefits over time.

Overall, Li et al have to be congratulated for further expanding the evidence on the added value of CPET variables out of peak $\dot{\text{V}}{\text{O}}_{2}$ and VE/VCO_2_ slope. We should take advantage of their message remembering to “put the finger” on O_2_ pulse as meaningful variable any time we wish to gain hemodynamic and prognostic insights when testing HFpEF.

## Funding support and author disclosures

The author has reported that they have no relationships relevant to the contents of this paper to disclose.
